# Subcutaneous Administration of Bortezomib in Combination with Thalidomide and Dexamethasone for Treatment of Newly Diagnosed Multiple Myeloma Patients

**DOI:** 10.1155/2015/927105

**Published:** 2015-09-06

**Authors:** Shenghao Wu, Cuiping Zheng, Songyan Chen, Xiaoping Cai, Yuejian Shi, Bijing Lin, Yuemiao Chen

**Affiliations:** Department of Hematology, Wenzhou Central Hospital, Dingli Clinical College of Wenzhou Medical University, Zhejiang 32500, China

## Abstract

*Objective*. To investigate the efficacy and safety of the treatment of the newly diagnosed multiple myeloma (MM) patients with the therapy of subcutaneous (subQ) administration of bortezomib and dexamethasone plus thalidomide (VTD) regimen.* Methods*. A total of 60 newly diagnosed MM patients were analyzed. 30 patients received improved VTD regimen (improved VTD group) with the subQ injection of bortezomib and the other 30 patients received conventional VTD regimen (VTD group).The efficacy and safety of two groups were analyzed retrospectively. *Results*. The overall remission (OR) after eight cycles of treatment was 73.3% in the VTD group and 76.7% in the improved VTD group (*P* > 0.05). No significant differences in time to 1-year estimate of overall survival (72% versus 75%, *P* = 0.848) and progression-free survival (median 22 months versus 25 months; *P* = 0.725) between two groups. The main toxicities related to therapy were leukopenia, neutropenia, thrombocytopenia, asthenia, fatigue, and renal and urinary disorders. Grade 3 and higher adverse events were significantly less common in the improved VTD group (50%) than VTD group (80%, *P* = 0.015). *Conclusions*. The improved VTD regimen by changing bortezomib from intravenous administration to subcutaneous injection has noninferior efficacy to standard VTD regimen, with an improved safety profile and reduced adverse events.

## 1. Introduction

Bortezomib, the first potent therapeutic proteasome inhibitor, has been suggested as a standard care in patients with newly diagnosed and relapsed multiple myeloma (MM) [1]. Bortezomib is associated with high efficacy response rate when it is used as induction therapy before high-dose therapy (HDT) plus autologous stem cell transplantation (ASCT) [2, 3]. Intravenous injection is the standard route of bortezomib administration; the recommended dose and schedule of bortezomib is 1.3 mg/m^2^ on days 1, 4, 8, and 11 of a 21-day cycle, for up to eight cycles, administered by 3–5/second intravenous (IV) bolus; this dose and schedule is active and well tolerated [4, 5]. However, IV administration requires repeated intravenous access or insertion of long-term central venous access devices and is usually associated with some serious adverse events [6].

Recently, two clinical trials have confirmed that subcutaneous (subQ) administration of bortezomib represents a good option to optimize the use of bortezomib for MM patients and results in a more convenient route that is at least as effective as the IV route [7, 8]. A phase I study conducted by French Francophone Myeloma Intergroup compared the pharmacokinetics and pharmacodynamics, safety and efficacy of IV, and subQ administration of bortezomib in patients with relapsed and/or refractory MM. The results demonstrated that subQ administration of bortezomib was possible because there were no differences in overall systemic availability and pharmacodynamic activity, toxicity profiles, and response rates in MM [7]. An international, multicenter, and randomized phase III study was then performed to confirm the safety and efficacy of this new route; 222 patients were randomly assigned to receive up to eight 21-day cycles of subcutaneous or intravenous bortezomib; the results confirmed that subQ bortezomib was not inferior to standard IV route, with even an improved safety profile and lower incidence of severe adverse events [8].

Bortezomib was approved for the treatment of MM in 2003, and since then several bortezomib-based combination therapies have developed. Regimens that have combined bortezomib with corticosteroids, alkylating agents, and immunomodulation drugs have resulted in high response rates [9]. The triplet combination of bortezomib and thalidomide plus dexamethasone (VTD) has proved to be a highly effective and well tolerated induction therapy for MM patients who were eligible for HDT-ASCT [10–12]. However, no relevant literatures were found regarding the subQ bortezomib-based VTD regimen as induction therapy for patients with MM. Therefore, the current single-center, retrospective study was designed to investigate the efficacy and safety of improved VTD regimen with the subQ administration of bortezomib in the treatment of the newly diagnosed MM patients.

## 2. Materials and Methods

### 2.1. Patients

A total of 60 patients with newly diagnosed MM from January 2009 to June 2013 who did not take part in a clinical trial were included in this study. According to practice guidelines at our center, patients were not excluded from VTD therapy on the basis of creatinine clearance rate or dialysis dependence. Patients with grade 2 or worse peripheral neuropathy were offered alternative therapy; 30 of them received improved VTD regimen (improved VTD group) with the subQ injection of bortezomib, and the other 30 patients received conventional VTD regimen (VTD group). The study was approved by the institutional review board of our hospital. Informed consents were obtained from the patients prior to this study.

### 2.2. Study Design

All the patients belonged to International Stage System (ISS) I–III in which transplantation patients were excluded or the patients refused receiving transplantation therapy. In both groups, all the patients were treated with VTD regimen as induction therapy. Bortezomib, at a dose of 1.3 mg/m^2^, on days 1, 4, 8, and 11, was administered by subcutaneous (improved VTD group) or intravenous injection (VTD group). Subcutaneous injections were administered at 2.5 mg/mL (3.5 mg bortezomib reconstituted with 1.4 mL normal saline) to limit the volume injected. Subcutaneous injection sites were the thighs or abdomen, which were rotated for successive injections. Intravenous injections were administered at a concentration of 1 mg/mL (3.5 mg bortezomib reconstituted with 3.5 mL normal saline) as a 3–5 s intravenous push. Oral thalidomide was given every day at a dose of 200 mg/d. Oral dexamethasone was given at a dose of 40 mg/d on days 1 through 4 and days 9 through 12. Each cycle was repeated every 21 d for up to 8 cycles. Treatment was suspended when drug-related grade 4 hematological toxic effects or grade 3-4 nonhematological toxic effects occurred.

### 2.3. Assessments

All patients were assessed for response and progression according to the international uniform response criteria for multiple myeloma (IMWG) [13] every 3 weeks. Baseline evaluations including physical examination, blood counts, hepatic and renal function tests, bone marrow aspirate and biopsy, serum and urine protein electrophoreses, and quantitation of serum immunoglobulin and urinary light chains and *β*_2_-MG were performed before each cycle. Interphase FISH were performed to identify cytogenetic abnormalities. Toxicities were evaluated according to National Cancer Institute Common Terminology Criteria for Adverse Events Version 3.0. Patients receiving at least two cycles of VTD regimen were included in the toxicity evaluation.

### 2.4. Statistical Analysis

SPSS version 17.0 software (Chicago, IL) was used for data analysis. The efficacy was evaluated by chi square test. Survival analysis was performed with life table and Kaplan-Meier survival curve. *P* value less than 0.05 was considered statistically significant.

## 3. Results

### 3.1. Patient Characteristics

A total of 60 patients with MM were recruited in this retrospective analysis. 30 patients received VTD therapy and the other 30 patients received improved VTD therapy. Their demographic and baseline characteristics are summarized in Table 1. Among these patients, 35 were males and 25 were females; the median age was 56 years (range, 31 to 72 years). IgG MM was found in 26 patients, IgA in 16 patients, IgD in 5 patients, and light chain MM in 13 patients. 12 patients were stage 1, 33 were stage II, and 15 were stage III. The baseline characteristics were similar in the two groups.

### 3.2. Efficacy

In both groups, patients received a median of six treatment cycles (range, four to eight). Overall remission (OR) after eight-cycle treatment was 73.3% in the VTD group (22 of 30 patients) and 76.7% in the improved VTD group (23 of 30 patients), including 4 patients (13.3%) getting complete remission (CR), 10 (33.3%) very good partial response (VGPR), and 8 (26.7%) partial remission (PR) in the VTD group and 3 patients (10%) getting CR, 11 (36.7%) VGPR, and 9 (30%) PR in the improved VTD group (Table 2). There was no statistical difference between the two groups (*P* > 0.05).

### 3.3. Prognosis

**Figure 1:**
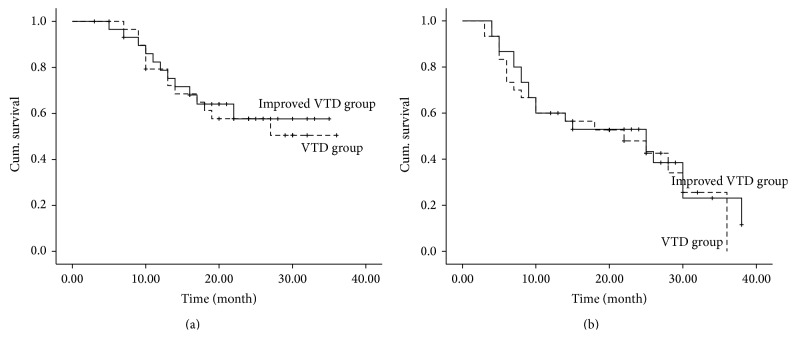
Kaplan-Meier curve of overall survival (a) and progression-free survival (b) after bortezomib and thalidomide plus dexamethasone (VTD) induction therapy.

After a median follow-up of 24 (range, 3–36) months, we noted no significant difference in 1-year estimate of overall survival (72% versus 75%, *P* = 0.848) and progression-free survival (median 22 months, 95% CI 7.16–36.8, versus 25 months, 95% CI 9.08–36.1; *P* = 0.725) between VTD group and improved VTD group (Figure 1).

### 3.4. Safety

All patients experienced at least one adverse event. The main toxicities related to therapy in the two groups included leukopenia, neutropenia, thrombocytopenia, asthenia, fatigue, and peripheral sensory neuropathy (Table 3). Most adverse events were grades 1-2. Grade 3 and higher adverse events were reported in 24 of 30 (80%) patients in the VTD group and 15 of 30 (50%) in the improved VTD group (*χ* ^2^ = 5.943, *P* = 0.015), with 8 (26.7%) and 3 (10%) discontinuing and 8 (26.7%) and 2 (6.7%) needing bortezomib dose reductions because of adverse events, respectively. Three of 30 (10%) patients in improved VTD group had one or more subcutaneous injection-site reaction reported, which resulted in a bortezomib dose modification in two (6.7%) patients (discontinuation or dose withholding). The most common reaction was injection-site erythema. No death related to therapy was reported in this study.

## 4. Discussion

In recent years, the outcome of MM patients has been significantly improved due to the discovery of novel antimyeloma agents together with a better knowledge of the pathophysiology of the disease. Among them, the proteasome inhibitor bortezomib (Velcade) represents an excellent drug that has quickly moved from the bench to the bedside and exhibits a powerful antimyeloma activity. Nowadays, bortezomib-based therapies are suggested as standards of care in patients with newly diagnosed and relapsed multiple myeloma [1]. In addition, abundant studies about the efficacy of bortezomib as a single agent or in combination with other agents in relapsed and/or refractory as well as in newly diagnosed myeloma patients have emerged, and all data have contributed to confirming bortezomib as one of the key drugs of the backbone treatment of myeloma patients [9].

The triplet combination of bortezomib and thalidomide plus dexamethasone (VTD) regimen was one of the highly effective and well tolerated induction therapies for MM patients. In our study, the overall response rate was 73.3% with VTD regimen therapy, including 13.3% CR, 33.3% VGPR, and 26.7% PR in newly diagnosed MM patients. Previous phase 3 study by the Italian Group for Adult Hematologic Diseases (GIMEMA) compared VTD with TD as induction therapy in newly diagnosed patients [14]. The results showed that VTD produced significantly higher response rates than TD both after induction (94% overall rate, including a 62% VGPR rate and a 32% CR/near-CR rate, versus 79% overall rate, including a 29% VGPR rate and a 12% CR/near-CR rate) and after transplantation (a 76% VGPR rate, including a 55% CR/near-CR rate, versus a 58% VGPR rate, including a 32% CR/near-CR rate). Combination of bortezomib with other immunomodulatory drugs and dexamethasone as induction therapy in newly diagnosed patients with MM also has been demonstrated in 2 studies of the combination of bortezomib, the thalidomide analog lenalidomide, and dexamethasone, which produced a 100% overall response rate, including a 75% VGPR rate and a 40% CR/near-CR rate [15, 16]. These results and ours demonstrate that VTD regimen is highly active and well tolerated as induction therapy in patients with MM.

The primary goal of the retrospective study was to compare the subQ bortezomib-based VTD regimen and conventional VTD regimen as induction therapy for patients with MM. Subcutaneous administration of bortezomib has been shown to be noninferior to the standard intravenous route of delivery in patients with relapsed multiple myeloma and has an improved systemic safety profile [7, 8]. Recently, subQ bortezomib-based regimen has emerged and is considered as a promising alternative to intravenous administration, particularly in patients with poor venous access or at increased risk of side-effects [17–19]. In this study, the improved VTD regimen by changing bortezomib from intravenous administration to subcutaneous injection showed noninferior efficacy to standard VTD regimen. We recorded similarity between groups across all efficacy endpoints, including rates of OR and CR and very good PR after eight cycles. This finding accords with previous results showing similar response rates of MM patients treated with improved bortezomib, adriamycin, and dexamethasone (PAD, 61.1%) with the subQ injection of bortezomib and conventional PAD regimen (57.1%) [17]. We also found that there were no significant differences in time to 1-year estimate of overall survival (72% versus 75%, *P* = 0.848) and progression-free survival (median 22 months, 95% CI 7.16–36.8, versus 25 months, 95% CI 9.08–36.1; *P* = 0.725) between VTD group and improved VTD group. Taken together, this study provided further information that subQ administration of bortezomib is feasible and could contribute to optimizing the management of bortezomib in the treatment of myeloma patients.

We also provided important findings about the toxic effects of bortezomib in the subcutaneous group and intravenous group. The main toxicities related to therapy in the two groups were leukopenia, neutropenia, thrombocytopenia, asthenia, fatigue, and peripheral sensory neuropathy. Subcutaneous administration had an improved systemic safety profile compared with intravenous delivery, with lower rates of grade 3 or higher adverse events, and with fewer bortezomib dose reductions and discontinuations because of adverse events. Subcutaneous administration also had acceptable local tolerability; only 3 patients developed one or more subcutaneous infection-site reactions reported as an adverse event, as resulted in a bortezomib dose modification in two patients. All of these results confirmed that subQ bortezomib-based VTD regimen was not inferior to IV route, with even an improved safety profile.

In conclusion, VTD is highly active and well tolerated induction therapy for patients with MM. The improved VTD regimen by changing bortezomib from intravenous administration to subcutaneous injection has noninferior efficacy to standard VTD regimen and may become the front-line therapy for the newly diagnosed MM patients. Further studies in larger populations and a long follow-up are warranted to confirm the result.

## Figures and Tables

**Table 1:** Patient demographics and baseline characteristics (*n* = 60).

Characteristic	VTD group (*n* = 30)	Improved VTD group (*n* = 30)	*P* value
Sex (male/female)	18/12	17/13	0.793
Median age (years, range)	54 (31–67)	57 (34–72)	0.712
Myeloma type			
IgG	12 (40%)	14 (46.7%)	0.602
IgA	9 (30%)	7 (23.3%)	0.559
IgM	2 (6.7%)	3 (10%)	0.640
Light chain	7 (23.3%)	6 (20%)	0.754
ISS stage			
I	6 (20%)	6 (20%)	1.000
II	18 (60%)	15 (50%)	0.436
III	6 (20%)	9 (30%)	0.371
Cytogenetics			
Diploid	15 (50%)	13 (43.3%)	0.605
Hyperdiploid	6 (20%)	7 (23.3%)	0.754
Nonhyperdiploid	6 (20%)	8 (26.7%)	0.542
Hypodiploid	3 (10%)	2 (6.7%)	0.640
Interphase FISH			
t(4;14)	15 (50%)	12 (40%)	0.436
del(17p13)	9 (30%)	14 (46.7%)	0.184
t(11;14)	6 (20%)	4 (13.3%)	0.488
Hemoglobin (g/L)	103 (71–144)	109 (73–159)	0.677
Albumin (g/L)	37.5 (22–47)	36 (24–43)	0.820
*β*_2_ microglobulin (mg/L)	3.9 (2.2–16.9)	4.3 (2.3–18.3)	0.754
Platelets (×10^9^/L)	243.4 (98.2–602.1)	251.7 (79.3–533.2)	0.501
Creatinine (mg/dL)	1.6 (0.4–3.7)	1.7 (0.2–4.1)	0.835

**Table 2:** Response to VTD regimen in each group.

Response (*n*, %)	After 8 cycles		
	VTD group (*n* = 30)	Improved VTD group (*n* = 30)	*P* value
OR	22 (73.3%)	23 (76.7%)	0.766
CR	4 (13.3%)	3 (10%)	0.688
VGPR	10 (33.3%)	11 (36.7%)	0.787
PR	8 (26.7%)	9 (30%)	0.774
MR	4 (13.3%)	4 (13.3%)	1.000
SD	3 (10%)	3 (10%)	1.000
PD	1 (3.3%)	0	0.313
Not evaluable	0	0	1.000

OR (CR + VGPR + PR): overall response; CR: complete response; VGPR: very good partial response; PR: partial response; MR: minimal response; SD: stable disease; PD: progressive disease; VTD: bortezomib and thalidomide plus dexamethasone.

**Table 3:** Incidence of adverse events related to VTD regimen in each group.

Events (*n*, %)	VTD group (*n* = 30)		Improved VTD group (*n* = 30)		*χ* ^2^	*P* ^*^
	All grades	Grade ≥ 3	All grades	Grade ≥ 3		
Leukopenia	27 (90%)	18 (60%)	22 (73.3%)	9 (30%)	5.455	**0.020**
Neutropenia	25 (83.3%)	13 (43.3%)	22 (73.3%)	12 (40%)	0.069	0.793
Thrombocytopenia	27 (90%)	19 (63.3%)	24 (80%)	16 (53.3%)	0.617	0.432
Anemia	15 (50%)	5 (16.7%)	12 (40%)	5 (16.7%)	0.000	1.000
Peripheral sensory neuropathy	17 (56.7%)	6 (20%)	10 (33.3%)	3 (10%)	1.176	0.278
Diarrhoea	11 (36.7%)	3 (10%)	10 (33.3%)	1 (3.3%)	1.071	0.301
Neuralgia	3 (10%)	1 (3.3%)	2 (6.7%)	0	1.107	0.313
Pyrexia	2 (6.7%)	0	1 (3.3%)	0	0.000	1.000
Nausea	18 (60%)	5 (16.7%)	10 (33.3%)	3 (10%)	0.577	0.448
Vomiting	14 (%)	4 (13.3%)	10 (33.3%)	3 (10%)	0.162	0.688
Asthenia	24 (80%)	5 (16.7%)	17 (56.7%)	3 (10%)	0.577	0.448
Constipation	12 (40%)	2 (6.7%)	9 (30%)	0	2.069	0.150
Fatigue	26 (86.7%)	11 (36.7%)	21 (70%)	4 (13.3%)	4.356	**0.037**
Weight decreased	3 (10%)	0	1 (3.3%)	0	0.000	1.000
Pneumonia	4 (13.3%)	0	2 (6.7%)	0	0.000	1.000
Eye disorders	2 (6.7%)	0	0	0	0.000	1.000
Renal and urinary disorders	21 (70%)	4 (13.3%)	13 (13.3%)	1 (3.3%)	1.964	0.161
Skin and subcutaneous tissue disorders	4 (13.3%)	1 (3.3%)	2 (6.7%)	0	1.107	0.313
Hepatobiliary disorders	6 (20%)	3 (10%)	4 (13.3%)	2 (6.7%)	0.218	0.640
Psychiatric disorders	1 (3.3%)	0	0	0	0.000	1.000

∗ shows that grade 3 and higher adverse events were compared by chi square test. VTD: bortezomib and thalidomide plus dexamethasone; improved VTD: subQ bortezomib and thalidomide plus dexamethasone.
